# From Primary Data to Ethnopharmacological Investigations on *Achillea erba-rotta* subsp. *moschata* (Wulfen) I.Richardson as a Remedy against Gastric Ailments in Valmalenco (Italy)

**DOI:** 10.3390/plants13040539

**Published:** 2024-02-16

**Authors:** Martina Bottoni, Giulia Martinelli, Nicole Maranta, Emanuela Sabato, Fabrizia Milani, Lorenzo Colombo, Paola Sira Colombo, Stefano Piazza, Enrico Sangiovanni, Claudia Giuliani, Piero Bruschi, Giulio Vistoli, Mario Dell’Agli, Gelsomina Fico

**Affiliations:** 1 Department of Pharmaceutical Sciences, University of Milan, Via Mangiagalli 25, 20133 Milan, Italy; martina.bottoni@unimi.it (M.B.); emanuela.sabato@unimi.it (E.S.); lorecolo.93@gmail.com (L.C.); pasico19@virgilio.it (P.S.C.); claudia.giuliani@unimi.it (C.G.); giulio.vistoli@unimi.it (G.V.); gelsomina.fico@unimi.it (G.F.); 2 Botanical Garden G.E. Ghirardi, Department of Pharmaceutical Sciences, University of Milan, Via Religione 25, 25088 Toscolano Maderno, BS, Italy; 3 Department of Pharmacological and Biomolecular Sciences “Rodolfo Paoletti”, University of Milan, Via Balzaretti 9, 20133 Milan, Italy; giulia.martinelli@unimi.it (G.M.); nicole.maranta@unimi.it (N.M.); stefano.piazza@unimi.it (S.P.); enrico.sangiovanni@unimi.it (E.S.); mario.dellagli@unimi.it (M.D.); 4 Department of Agricultural, Environmental, Food and Forestry Science and Technology, University of Florence, Piazzale delle Cascine 18, 50144 Florence, Italy; piero.bruschi@unifi.it

**Keywords:** traditional medicine, *Achillea erba-rotta* subsp. *moschata* (Wulfen) I.Richardson, molecular docking, anti-inflammatory activity, *Helicobacter pylori*

## Abstract

(1) Background: Within the framework of the European Interreg Italy–Switzerland B-ICE & Heritage project (2018–2022), this study originated from a three-year ethnobotanical survey in Valmalenco (Sondrio, Italy). Following a preliminary work published by our group, this research further explored the folk therapeutic use of *Achillea erba-rotta* subsp. *moschata* (Wulfen) I.Richardson (Asteraceae) for dyspepsia disorders, specifically its anti-inflammatory potential at a gastrointestinal level. (2) Methods: Semi-structured interviews were performed. The bitter taste was investigated through molecular docking software (PLANTS, GOLD), while the anti-inflammatory activity of the hydroethanolic extract, infusion, and decoction was evaluated based on the release of IL-8 and IL-6 after treatment with TNFα or *Helicobacter pylori*. The minimum inhibitory concentration and bacterial adhesion on the gastric epithelium were evaluated. (3) Results: In total, 401 respondents were interviewed. Molecular docking highlighted di-caffeoylquinic acids as the main compounds responsible for the interaction with bitter taste receptors. The moderate inhibition of IL-6 and IL-8 release was recorded, while, in the co-culture with *H. pylori*, stronger anti-inflammatory potential was expressed (29–45 μg/mL). The concentration-dependent inhibition of *H. pylori* growth was recorded (MIC = 100 μg/mL), with a significant anti-adhesive effect. (4) Conclusions: Confirming the folk tradition, the study emphasizes the species’ potentiality for dyspepsia disorders. Future studies are needed to identify the components mostly responsible for the biological effects.

## 1. Introduction

Collecting absolute field data concerning the therapeutic use of plants represents the core of ethnobotany. The traditional knowledge spontaneously given by the pool of respondents defines the local biocultural heritage and in turn reflects the human–environment relationship. This relationship becomes tangible through the safeguarding of traditional practices related to the sustainable use of plant resources [1,2,3].

In mountain areas, ethnobotanical knowledge is currently endangered, largely due to the ongoing climate and socioeconomic changes. Higher temperatures and the decline in snow cover, among other factors, can negatively impact alpine biodiversity [4], as well as the availability of autochthonous plant species, thus potentially affecting traditional knowledge in the long term. Simultaneously, the depopulation of the mountains over the past few decades has generated steady outgoing flows of people and, ultimately, the increasing neglect of local traditions [5]. The loss of these traditions could result in the declining ability of local communities to manage and preserve their ecosystems [5] and has direct consequences for the use of biological resources by future generations [6,7]. Within the context of the European Interreg Italy–Switzerland B-ICE & Heritage project (ID 63143), a three-year ethnobotanical survey was conducted in Valmalenco (Italy, Lombardy, Province of Sondrio), a valley located in the Italian Central Alps. The valley is made up of five municipalities (Chiesa in Valmalenco, Caspoggio, Lanzada, Spriana, and Torre di Santa Maria) and has a history of isolation, beginning in the second half of the 18th century [8].

Previous ethnobotanical surveys have been carried out in the alpine and pre-alpine areas surrounding Valmalenco [9,10]. None of these have used raw primary data to discuss the relationship between humans and plants, giving instead relevance to ethnobotanical indexes as a tool for the selection of medicinal plants [11]. In this context, we previously focused on a multidisciplinary investigation of *Achillea erba-rotta* subsp. *moschata* (Wulfen) I.Richardson (syn. *Achillea moschata* Wulfen [12]), starting with the analysis of the absolute data collected in Chiesa in Valmalenco. Paying attention to the species’ use in folk medicine to treat gastrointestinal ailments, the work brought an element of novelty by proposing an interdisciplinary study of the traditional aqueous preparation. Morpho-anatomical and histochemical investigations were performed on the traditional herbal drug, along with phytochemical and cell-based *in vitro* surveys. The work represented the first effort to correlate the traditional use of a hot aqueous extract against inflammatory-based gastric disorders with its chemical polyphenolic composition and antioxidant/anti-inflammatory cross-talk abilities [13]. Consequently, the study presented herein represents a further investigation aimed at deepening the overall primary data documented in Valmalenco for *A. erba-rotta* subsp. *moschata*, to obtain even more raw information on the species’ potential for the treatment of dyspepsia disorders.

Nowadays, functional dyspepsia represents one of the most common gastrointestinal disorders, affecting more than 10% of the population all over the world. The symptoms can be severe and disabling, reducing the individual’s quality of life. *Helicobacter pylori* is a leading cause of chronic gastric inflammation, generally associated with gastritis, adenocarcinoma, and functional dyspepsia. The activation of the NF-κB pathway mainly contributes to the inflammatory phenotype observed in *H. pylori* infection in humans and experimental models. The major inflammatory signal is the release of IL-8, whose levels correlate with *H. pylori* infection and gastric disease severity [14,15,16,17], but other mediators, such as IL-6 and TNFα, also play pivotal roles during disease progression [18]. In humans, the sole presence of *H. pylori* in the stomach is not able to have a clinical impact on the symptoms of patients with functional dyspepsia; however, the duodenal inflammation associated with *H. pylori* infection significantly enhances the severity of the dyspeptic symptoms [19]. The first-line pharmacological treatment consists of proton-pump inhibition or *H. pylori* eradication, which provides significant improvements in symptoms [20]. However, this approach often is not enough. Neuromodulating agents are commonly used in clinical practice, but only tricyclic antidepressants (TCAs) are mentioned in the European and American guidelines [21]. Moreover, low compliance has been documented, due to high therapy costs, among others [22]. Considering the variety of mechanisms involved, multicomponent herbal extracts have been studied as a source of many substances potentially able to act with different mechanisms [23,24,25].

Considering this premise, and according to our previous study, this work was aimed at (1) recording the traditional uses of *A. erba-rotta* subsp. *moschata* in Valmalenco and analyzing the collected information by using a qualitative and quantitative approach; (2) thoroughly investigating the strong bitter taste of the traditional preparation by means of molecular docking analysis, as an element of novelty; (3) performing biological assays, according to the traditional use of *A. erba-rotta* subsp. *moschata*, aimed at investigating its ability to impair *H. pylori* growth and *H. pylori*-induced inflammation in human gastric epithelial GES-1 cells co-cultured with *H. pylori*. Since TNFα is released by monocytes/macrophages and epithelial cells during infection [26,27], the extracts’ ability to reduce the release of this cytokine was assessed as well.

## 2. Results and Discussion

### 2.1. Ethnobotanical Investigation: The Species and Its Uses

A total of 401 respondents, aged between 11 and 97, were interviewed in Valmalenco between June 2019 and April 2022. The pool of informants included 278 women and 123 men. Most of them (331; 82.5% of the total sample) reported at least one use of *A. erba-rotta* subsp. *moschata* (locally known as “*erba iva*”, “*aneda*”, “*daneda*”, or “*taneda*”) for a total number of 594 citations. The mean number of citations for this species was 1.79 ± 0.70, with a number of reported uses ranging from one (116 informants) to five (one informant). The reported use sectors were medicinal (277 informants; 343 citations), food (197; 225), and veterinary (25; 26).

#### 2.1.1. Medicinal Sector

Regarding the medicinal sector, the inflorescences represented the main used plant part (242 citations), followed by the whole epigeal part (74), mostly prepared as infusions (309 citations). The highest number of citations was recorded for digestive tract disorders (299 citations; 80% of the total citations in the medicinal sector). In particular, the local folk medicine described the decoction as a strong stomachic and eupeptic (269 citations) and for stomach pain (35). Often, the discomfort was associated with dyspepsia resulting from abundant meals, sometimes referring to congestion: “with that, it either goes up or down” (“*con quella, o va su o va giù*”), “it is a lifesaver” (“*è un salvavita*”), “it is extraordinary” (“*è portentosa*”), “it is very bitter and powerful” (“*è amarissima e molto potente*”), “the bitter taste is the good effect” (“*l’amaro è l’effetto buono*”), “it is a medicinal herb, 3–4 flowers and it helps me digest” (“*è un’erba medicinale, 3–4 fiorellini e mi fa digerire*”). For the complete records in Italian, please see Table S1 of the Supplementary Materials. However, in some cases, the problem was associated with pathological conditions such as gastritis and ulcers (nine citations) and gastric reflux (18), so much so that some informants stated that their physician suggested to take a cup of “*erba iva*” along with the pharmacological therapy, with the belief that “it never hurts to drink it”. Nevertheless, the general warning was not to use an excessive number of flower heads, not due to the very bitter taste but due to the risk of side effects: “however, you must not overdo it, too many flowers cause stomach ache”, “very few flowers are enough to have an effect”, “it causes me tachycardia”. Its digestive power was previously recorded by other ethnobotanical studies carried out in the alpine and pre-alpine regions [28,29,30], specifically in the valleys surrounding Valmalenco ([9,31] and the literature within), but none of these studies focused on an in depth-analysis of the primary data concerning the use of the species as a preventive or symptomatic agent. In fact, some informants in Valmalenco stated that the decoction must be used only when needed, while others advised to drink it every evening, without a specific therapeutic need. Considering this, the anti-inflammatory action emphasized by the locals, along with the powerful bitter taste and the frequency of use, allowed us to define a set of interrelated factors that could indicate the potential effectiveness of the traditional preparation.

The majority of informants (98.0%; 338 citations) reported to have used the plant “personally”, while 97.9% (337) stated that they still used it. Local people reported that the use of the species in the past was “very frequent” (343, 100% of the cases), while, at the time of the study, it was “very frequent” in 34.9% (120) and “quite frequent” in 67.3% of the cases (n = 231). This distinction was not linked to the loss of traditional knowledge but to the greater difficulty in finding the species [8].

#### 2.1.2. Food and Veterinary Sectors

The most cited category for food uses was liquor (197 citations; 87.5% of the total citations in food sector), followed by seasoning (21; 9.3%) and non-alcoholic beverages (6; 2.7%). The inflorescences represented the main used plant part (164 citations), followed by the epigeal part (61). All citations concerning the use of *A. erba-rotta* subsp. *moschata* as a liquor were characterized as “personal” and “ongoing”. Its preparation consists of macerations in grappa or 95% ethanol of inflorescences (147 citations) or the epigeal part (50), mixed with a syrup made with sugar and water. The importance of *A. erba-rotta* subsp. *moschata* in the veterinary sector referred mainly to the use of the plant decoction or infusion to improve digestion or to treat intestinal swelling in animals (in particular, cattle).

### 2.2. A Shared Cultural Heritage

We expected women to know more about the medicinal uses than men, because women in alpine areas represented the primary healthcare providers for the family until the recent past [31]. However, the results of the Mann–Whitney U Test showed that both female and male informants shared the same traditional knowledge of *A. erba-rotta* subsp. *moschata* (Table 1) and that the gendered division of labor had no effect on the uses of this plant. When analyzing the number of reported uses by age, we observed that 57% of the 331 informants were ≥60 years old (189 informants); of these, 181 were aged between 61 and 80. Furthermore, 40 of the informants were over 80. One hundred and forty-two (43%) informants were ≤60 years old—more precisely, between 18 and 60. The Spearman test showed that ethnobotanical knowledge increased significantly with age (R = 0.131; *p* < 0.05), suggesting that older individuals know more uses of the plant than those belonging to the younger group, and that this cultural heritage still survives in older generations (Table 1). It should be noted that this age-related increase was statistically significant only for medicinal uses (R = 0.135; *p* < 0.05) (Table 1). Several studies have shown that traditional knowledge is strongly subject to the risk of rapid erosion, due to the influence of modern culture and education systems [31] and to urbanization processes [32], causing a lack of interest in young people regarding ethnobotanical uses. However, this did not seem to be true for all the uses of *A. erba-rotta* subsp. *moschata* reported by the respondents; for example, the production and use of the liquor were known even by young informants. This finding is consistent with Soukand et al. [33] and Egea et al. [34], who showed that homemade alcoholic beverages are well known and practiced through the generations. When we considered the whole set of collected data, no statistically significant differences were observed among the studied communities (Table 1); statistically significant differences were detected between CHI and LAN in the knowledge of medicinal uses (Table 1). Specifically, CHI informants were more knowledgeable about the sedative uses of the plant.

All these findings suggest that *A. erba-rotta* subsp. *moschata* is an integral part of the community’s identity, contributing towards a shared cultural heritage based on the respect of the local traditions and the conservation of the alpine habitats. The daily relationship that *malenca* people developed over time towards this species did not emerge for other plants. Every house in Valmalenco was adorned with small bunches of air-dried flower heads of *A. erba-rotta* subsp. *moschata*, hanging upside down from kitchens or wooden beams, and it represented, for the *malenca* people, a source of pride and awareness of their own cultural tradition: “I don’t know the *erba iva*, I know the *daneda*”, “everyone uses it in Valmalenco”, “*erba iva* has never been lacking in our house”, “this is the manner here, you have to bring them together and let them dry”. Since ancient times, the collection of the plant’s inflorescences has been an annual appointment for the inhabitants of the valley. High mountain expeditions are experienced as an opportunity for family gathering and sharing. Both men and women, still able to reach its altitudes, were concerned about recovering the plant, including for those people who were no longer able to reach and collect it or for family members who no longer lived in the valley but remained attached to their traditions. Furthermore, this event represented a source of extreme care and attention among the local people towards the natural environment. The collection of the species was never indiscriminate in terms of the period, quantity, and methods. The inflorescences were always harvested during the balsamic period (normally reached in July/August based on altitude), only in quantities suitable to satisfy the family’s needs, cutting the stem at 2–3 cm from the ground with a small sickle or with scissors. Comments such as “the plant should never be torn off”, “you have to cut it more or less at this height, moving the flowers”, “you must always leave the roots, if you want to find it again next year”, and “don’t pick it up here, my neighbor always comes here” reflected the attention and care towards the natural environment, as well as the social network that this plant was able to create. In the past, the collection and sale of the plant represented a source of livelihood for the local community, so much so that the inflorescences were also sold to pharmacies to produce galenic preparations: “it was the treasure that made survival possible”. Recently, the collection of the plant has taken on a different meaning, aimed at personal healthcare and social relations. Despite the higher altitudes at which it is found, the local community’s need to harvest the species has never abated, and this accounts for the deep radicalization of its traditional use.

### 2.3. Molecular Docking Analysis

Bitter taste receptors belong to a group of GPCRs called type 2 taste receptors (TAS2Rs) [35,36,37]. TAS2Rs were discovered in an ever-growing number of extra-oral tissues. Their presence in the internal organs could be linked to their involvement in the regulation of different physiological processes, thus becoming putative targets for pharmacological treatment [38,39]. In the gastrointestinal tract, TAS2Rs are involved in the regulation of metabolic function, hunger/satiety modulation, digestion, and pathogen defense reactions. Among the best investigated processes, bitter compounds induce the secretion of peptide hormones (i.e., GLP-1, CCK, ghrelin), stomach acid secretion, the regulation of intestinal motility, and the defense against pathogenic organisms, such as helminths and bacteria [40]. Considering the purported health benefits associated with the bitter taste of *A. erba-rotta* subsp. *moschata,* we performed molecular docking studies to elucidate the ability of the TAS2R46 receptors to accommodate the 31 polyphenols identified in our previous work [13]. A preliminary redocking study was performed with two different docking algorithms in order to evaluate the ability of the software to reproduce the experimental pose of the co-crystalized compound strychnine, a toxic bitter alkaloid able to activate the TAS2R46-mediated G protein gustducin signaling pathway [41]. Subsequently, docking analyses were carried out with the aim to understand which phytocomplex components were more able to bind the receptor and had a potential role in the physiopathological gastrointestinal processes described by the *malenca* people.

### Preliminary Redocking Study and Docking Analysis

The main interactions between the receptor and the docked ligand were preserved in the resolved complex. The binding pocket seems to be a wide-open funnel, composed of residues from TM2, TM3, TM5, and TM7. Trp88 and Glu265 play a key role in the recognition of strychnine; the former interacts via pi–pi stacking, while the Glu establishes an ion pair with the protonated amine group of strychnine. Trp88 has a crucial role, since a mutagenesis assay showed that the mutation in Ala abolished the strychnine activity. The redocking analysis was performed with the software programs PLANTS (v. 1.2) and GOLD (v. 5.8.1), and they gave good results, as shown by the RMSD values between the experimental pose of the strychnine and the docked one, which were, in both cases, less than 2Å. PLANTS docking gave an RMSD of 1.03Å based on all atoms, while GOLD docking gave an RMSD value of 0.90Å based on all atoms.

**Figure 1:**
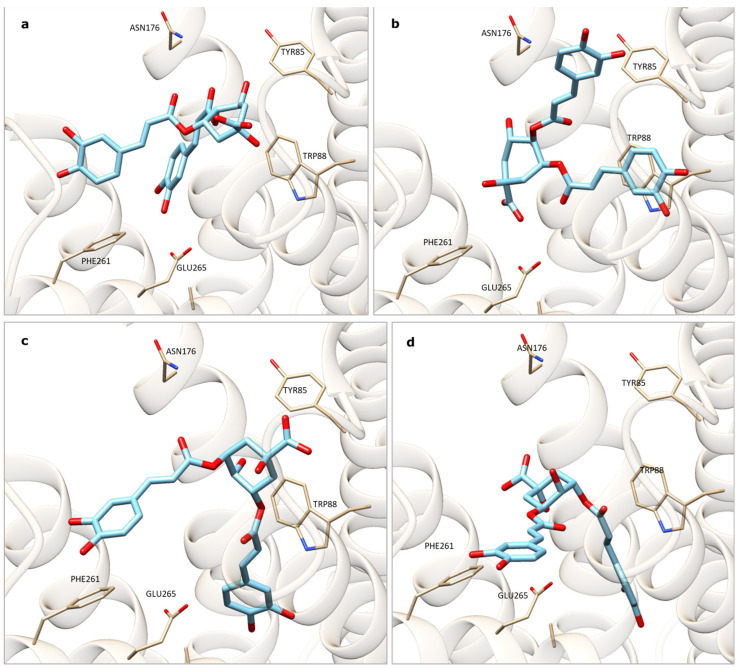
(**a**,**b**) Interaction of 4,5-O-dicaffeoylquinic acid with TAS2R46 by means of v. 1.2 GOLD (**a**) and v. 5.8.1 PLANTS (**b**) software. (**c**,**d**) Interaction of 3,5-O-dicaffeoylquinic acid with TAS2R46 by means of GOLD (**c**) and PLANTS (**d**) software.

Subsequently, the docking analysis involved the 31 extract constituents identified in *A. erba-rotta* subsp. *moschata* through the phytochemical analysis [13], and the consensus results were summarized in a table in descending order of interaction (Table 2). The results reasonably suggested that 4,5-O-dicaffeoylquinic acid and 3,5-O-dicaffeoylquinic acid, as well as luteolin-7-O-(6″-malonylglucoside) and isorhamnetin-3-O-rutinoside, were among the main compounds responsible for the decoction’s strong bitter taste, since the interactions of these molecules with the receptor were established with Trp88 and Glu265 (as occurred for strychnine) and Tyr85, and, in some cases, we observed pi stacking with Phe261 (Figure 1).

In fact, polyphenols represent a group of molecules that show bitterness properties and are able to bind to various TAS2Rs among the 25 human isoforms. Their specificity of action is linked to their molecular structure. The structural requirements for flavonoids to activate the bitter receptors seem to be linked to the presence of two or three hydrogen bond donor sites, one hydrogen bond acceptor site, and two aromatic ring structures, one of which must be hydrophobic, thus allowing us to predict the potential bitterness of these bioactive molecules [42]. On the other hand, epicatechins upregulate ghrelin release through gut TAS2R activation, apigenin displays anti-inflammatory effects in respiratory tract infections through an activation mechanism, and methoxy flavones act at the same level as antagonists, thus opening up new perspectives on phytotherapy and health strategies [43,44,45]. To this purpose, caffeic acid derivatives were recently studied as some of the biologically active metabolites of propolis, showing a large spectrum of action. The isolated caffeic acid phenethyl ester showed multiple pharmacological effects—for example, at a gastrointestinal level, where it suppressed the production of pro-inflammatory cytokines and enhanced the epithelial barrier function, inhibiting the NF-κB pathway’s overreaction in models of ulcerative colitis. These aromatic acids were also able to act as anti-inflammatory agents within the framework of autoimmune diseases, cancer, and respiratory tract-related diseases [46,47]. In light of this, along with healthy lifestyles and gastroprotective drugs, more clinical evidence is needed to underline the potential contribution in complementary therapy of dietary polyphenols and their metabolites at a gastrointestinal level [48].

**Table 2:** Results of the docking analysis performed for the 31 components identified in *A. erba-rotta* subsp. *moschata* [13].

Ligand	Peak Number	PLANTS Rank	GOLD Rank	Consensus ^1^
4,5-O-Dicaffeoylquinic acid *^,^**	11, 13, 19	4	2	6
3,5-O-Dicaffeoylquinic acid *^,^**	11, 13, 19	2	6	8
Luteolin-7-O-(6″-malonylglucoside)	20	1	11	12
Isorhamnetin-3-O-rutinoside *^,^**	14	15	1	16
Luteolin-C-glucoside	4	12	9	21
Cosmosiin	15	8	20	28
Luteolin-7-O-glucoside *^,^**	8	7	22	29
Rutin	6	17	14	31
Isorhamnetin-3-O-glucoside *^,^**	12, 16	25	8	33
Vicenin-2	3	16	18	34
Apigenin-7-O-(6″-malonylglucoside)	22	6	28	34
Mearnsetin-hexoside	9, 10	23	15	38
Syringetin-3-O-glucoside	17	32	10	42
Schaftoside	5	19	23	42
5-O-Caffeoylquinic acid *^,^**	1	10	32	42
Kaempferol-3-O-glucoside *	7	31	13	44
Desmethoxycentaureidin	27	22	24	46
Eupatolin	21	37	12	49
Isoorientin-7-methyl-ether	18	20	30	50
Chrysoeriol	26	24	27	51
Chlorogenic acid	2	18	38	56
Jaceidin	31	38	21	59
Quercetin-3,3′-dimethyl-ether	29	36	26	62
Hispidulin	26	29	35	64
Luteolin	23	28	36	64
Axillarin	24	40	25	65
Apigenin	25	26	40	66
Isorhamnetin	28	33	34	67
6-Hydroxykaempferol-3,6-dimethylether	30	39	29	68
Isorhamnetin-O-hexoside	12, 16	34	39	73

^1^ The consensus is defined by the sum of the positions in the two software programs: the highest is the sum; the worst is the interaction of the molecule with the receptor. * [11]; ** [49].

### 2.4. Pharmacological Investigation

Following the ethnobotanical approach and the traditional use that emerged for *A. erba-rotta* subsp. *moschata*, we focused on the investigation of the potential therapeutical properties of different *A. erba-rotta* subsp. *moschata* extracts (decoction, infusion, hydroethanolic extract) against gastric inflammatory disorders, mostly due to the presence of *H. pylori*. The infusion was tested as a viable alternative to the decoction, because, in some of the valleys surrounding Valmalenco, the use of the infusion was mentioned as well [9]. We investigated also the hydroalcoholic extract, as these types of preparations are more common at a production and commercial level.

The infusion and decoction were prepared according to the ethnobotanical survey and compared with an hydroethanolic extract. In the first set of experiments, gastric cells were challenged with TNFα or *H. pylori* as stimuli to mimic the gastric inflammatory conditions of the epithelium and dyspepsia, whereas the second part of the pharmacological approach was devoted to testing the antibacterial and anti-adhesive effects of the extracts. None of the extracts under study showed signs of cytotoxicity in the MTT assay in the concentration range of 10–200 μg/mL (Figure S1).

#### 2.4.1. Inhibition of TNFα-Induced IL-8 and IL-6 Release and NF-κB Activity in Human Gastric Epithelial GES-1 Cells

**Figure 2:**
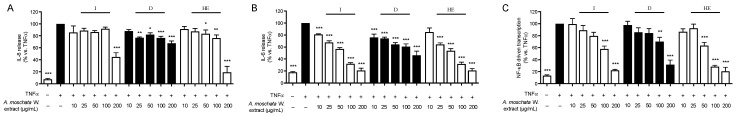
Effect of *A. erba-rotta* subsp. *moschata* extracts on TNFα-induced inflammation in GES-1 cells. GES-1 cells were treated for 6 h with TNFα (10 ng/mL) in addition to extracts; IL-8 and IL-6 (panel (**A**) and (**B**), respectively) were measured by ELISA assay, while the activation of NF-κB (panel (**C**)) was measured by luciferase assay. EGCG (20 μM) was used as reference compound to inhibit IL-8 release (−86.23%) and IL-6 release (−71.02%), while apigenin (20 μM) was used as reference compound able to inhibit NF-κB-driven transcription (−90.14%). Results are expressed as the mean ± SEM (at least three experiments) of the relative percentage in comparison to stimulus (black bar), to which the value of 100% was arbitrarily assigned. * *p* <0.05, ** *p* < 0.01, and *** *p* < 0.001 vs. stimulus. I, *A. erba-rotta* subsp. *moschata* infusion; D, *A. erba-rotta* subsp. *moschata* decoction; HE, *A. erba-rotta* subsp. *moschata* hydroethanolic extract.

During *H. pylori* infection, a variety of pro-inflammatory mediators are released by gastric epithelial cells and macrophages, particularly cytokines and chemokines (i.e., IL-6, TNFα, and IL-8). The release of TNFα by gastric epithelial cells and macrophages feeds the inflammatory process in the gastric mucosa, activating the cytokine cascade. To investigate the activity of *A. erba-rotta* subsp. *moschata* extracts in modulating the inflammatory process, GES-1 cells were treated for 6 h with TNFα as a stimulus and the extracts; then, IL-8 or IL-6 release was measured through an ELISA assay. The results are summarized in Figure 2.

All the extracts showed the moderate inhibition of IL-8 release (Figure 2, panel A), with an IC_50_ above 100 μg/mL (Table 3). Of note, the infusion showed 60% inhibition of TNFα-induced IL-8 release at 200 μg/mL. The most active extract was the hydroethanolic one, with an IC_50_ of 123.7 μg/mL.

All the extracts showed better activity in inhibiting IL-6 release in a concentration-dependent manner (Figure 2, panel B), with the IC_50_ ranging from 33 to 85 μg/mL (Table 3). The aqueous extracts showed the statistically significant inhibition of IL-6 release already at the lowest concentration tested (10 μg/mL), but the infusion showed a lower IC_50_ with respect to the decoction and comparable to the hydroethanolic extract.

Since NF-κB orchestrates the expression and release of IL-8 and IL-6, which, in turn, exacerbate the gastric phlogistic processes, we investigated the effects of the extracts on TNFα-induced NF-κB-driven transcription in gastric epithelial GES-1 cells. All the extracts impaired NF-κB-driven transcription in a concentration-dependent manner, with an IC_50_ in the low micromolar range (52.0–89.5 μg/mL), thus explaining, at least in part, the effects observed on IL-8 and IL-6 release (Figure 2, panel C).

We previously investigated the preventive effects of *A. erba-rotta* subsp. *moschata* aqueous and methanolic extracts on the NF-κB pathway induced by TNFα [13]. However, the effect in our previous paper was observed during the pre-treatment of cells with the extract, whereas, here, the effect was demonstrated during co-treatment. Thus, this is the first study that demonstrates the NF-κB impairment in human gastric non-tumoral epithelial cells by *A. erba-rotta* subsp. *moschata* extracts.

#### 2.4.2. Inhibition of *H. pylori*-Induced IL-8 and IL-6 Release and NF-κB Activity in Human Gastric Epithelial GES-1 Cells

**Figure 3:**
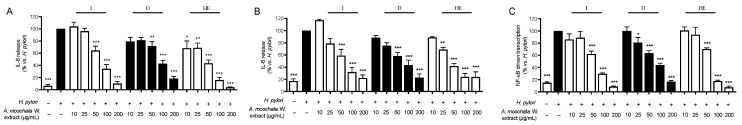
Effect of *A. erba-rotta* subsp. *moschata* extracts on *H. pylori*-induced inflammation in GES-1 cells. GES-1 cells were treated for 6 h with *H. pylori* (ratio 50:1, bacteria:cell) in addition to extracts; IL-8 and IL-6 (panel (**A**) and (**B**), respectively) were measured by ELISA assay, while the activation of NF-κB (panel (**C**)) was measured by luciferase assay. EGCG (50 μM) was used as reference compound to inhibit IL-8 release (−72,73%) and IL-6 release (−69.67%), while apigenin (50 μM) was used as reference compound to inhibit NF-κB-driven transcription (−90.27%). Results are expressed as the mean ± SEM (at least three experiments) of the relative percentage in comparison to stimulus (black bar), to which the value of 100% was arbitrarily assigned. * *p* < 0.05, ** *p* < 0.01, and *** *p* < 0.001 vs. stimulus. I, *A. erba-rotta* subsp. *moschata* infusion; D, *A. erba-rotta* subsp. *moschata* decoction; HE, *A. erba-rotta* subsp. *moschata* hydroethanolic extract.

Another set of experiments was devoted to the evaluation of the anti-inflammatory effect of the extracts under study through a more sophisticated and complex approach, which was the co-culture *H. pylori*-GES-1 cells. It has been widely reported that *H. pylori* is able to increase the production of several cytokines, including IL-8 and IL-6, and activate NF-κB signaling. Thus, the effect of the three extracts on these inflammatory parameters induced by the pathogen was evaluated. Surprisingly, the effect on IL-8 release was more pronounced when cells were challenged with *H. pylori* than TNFα. In particular, all the extracts showed the concentration-dependent inhibition of cytokine release, with an IC_50_ ranging from 35.2 to 62.7 μg/mL. The hydroethanolic extract appeared to be around twice as effective as the aqueous extracts (Figure 3, panel A). The effect on *H. pylori*-induced IL-6 release in GES-1 cells was even better, with an IC_50_ from 29 to 45 μg/mL. The two aqueous extracts showed comparable effects on this parameter (Figure 3, panel B). The effects were found to be ascribed to the impairment of the NF-κB pathway, since all the extracts showed comparable inhibitory effects and IC_50_s to those reported for IL-8 and IL-6 release (Figure 3, panel C). Table 3 summarizes the IC_50_s calculated for all the parameters.

#### 2.4.3. Antibacterial and Anti-Adhesion Activity

**Figure 4:**
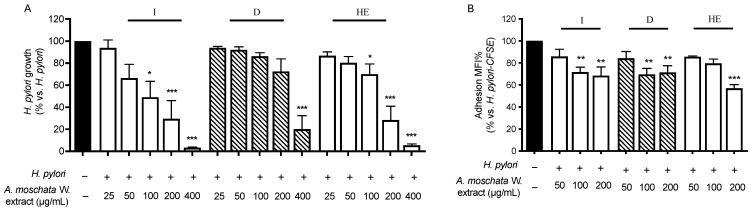
Effect of *A. erba-rotta* subsp. *moschata* extracts on *H. pylori* growth and adhesion to GES-1 cells. *H. pylori* (OD = 0.1) was treated for 72 h with extracts, and the rate of bacterial growth was measured as optical density (600 nm) using a photometer. Tetracycline (0.125 μg/mL) was used as reference compound. Results are expressed as the mean ± SEM (at least three experiments) of the relative percentage in comparison to *H. pylori* growth (black bar), to which the value of 100% was arbitrarily assigned (panel (**A**)). GES-1 cells were treated for 1 h with *H. pylori*–CFSE (ratio 50:1, bacteria:cell), in addition to extracts, and the bacterial adhesion to cells was measured as fluorescence intensity using a cytofluorimeter. Procyanidin A2 (500 μM) was used as reference compound (−83.32% adhesion inhibition). Results are expressed as the mean ± SEM (at least three experiments) of the relative percentage in comparison to *H. pylori* infection (black bar), to which the value of 100% was arbitrarily assigned (panel (**B**)). * *p* < 0.05, ** *p* < 0.01, and *** *p* < 0.001 vs. *H. pylori*. MIC, minimum inhibitory concentration; MFI%, median fluorescence intensity, CFSE, carboxyfluorescein succinimidyl ester; I, *A. erba-rotta* subsp. *moschata* infusion; D, *A. erba-rotta* subsp. *moschata* decoction; HE, *A. erba-rotta* subsp. *moschata* hydroethanolic extract.

Following the traditional use of *A. erba-rotta* subsp. *moschata* as a remedy to alleviate gastric ailments, promote digestion, and improve dyspeptic symptoms, we focused more in depth on the efficacy of the extract against *H. pylori* growth and adhesion on the gastric epithelium. The results are reported in Figure 4, panel A. Of note, only the infusion and hydroethanolic extract showed the concentration-dependent inhibition of *H. pylori* growth, with an MIC of 100 μg/mL for both the extracts and an MIC_50_ of 95 and 129 μg/mL, respectively. In contrast, the inhibitory effect of the decoction was evident only at the highest concentration tested (400 μg/mL). These findings prompted us to speculate that the active compounds able to inhibit *H. pylori* growth may be unstable at the high temperatures typical of the decoction procedure. Since the first step of *H. pylori* invasion is adhesion to gastric epithelial cells, the effect of the extracts in modulating adhesion was investigated as well. The aqueous extracts showed significant anti-adhesion effects at 100 and 200 μg/mL (around 30–35% inhibition), whereas the hydroethanolic extract was effective only at 200 μg/mL (around 40% inhibition) (Figure 4, panel B).

Taken together, our results demonstrate, for the first time, that the aqueous extracts of *A. erba-rotta* subsp. *moschata* impair *H. pylori* growth and its invasion towards the gastric epithelium; moreover, the extracts inhibited the pro-inflammatory processes induced by the presence of the pathogen.

## 3. Materials and Methods

### 3.1. Ethnobotanical Research

The ethnobotanical survey was carried out in Valmalenco (Italy, Lombardy, Province of Sondrio), in the municipalities of Chiesa in Valmalenco, Caspoggio, Lanzada, Spriana, and Torre di Santa Maria, between June 2019 and April 2022. The informants’ selection started with the identification of respondents able to create a social network within the local community and amplify the number of people that the researchers were able to access for interviews (survey’s intermediaries). In addition, we identified those who showed a wide heritage of traditions and the current application of traditional knowledge (key informants). Semi-structured interviews were conducted in Italian with some local dialectal inflections, without the help of interpreters. Individual or small group interviews (3–5 people) were favored, in the presence of at least two researchers. In this way, greater control over the information flow was guaranteed, as well as the correct annotation of the primary data and the establishment of an optimal degree of empathy. The interviews were conducted mainly in an open environment and, when it was not possible to meet the informant outdoors (i.e., people no longer able to move because of their age), the interview was conducted in the domestic environment. In this case, it was possible to evaluate the role of the plant in people’s daily lives, noting its presence or absence in the home environment, the care given to the correct preservation of the plant, the family roles, and the emotions associated with it. The interviews were approached with inclusivity, patience, and intellectual humility. The researcher always described the interview circumstances as a “light conversation” or “chat” (in Italian, they described it with the word “*chiacchierata*”), automatically bypassing hierarchical misunderstandings or refined languages that could recall more formal environments. Finally, tales of plant species were always linked to personal life stories, and, for this reason, the scientific documentation of the data required a sensitive and renewed approach to each new interview, complying with the wishes and availability of the informant. Data were collected and subsequently analyzed according to Bottoni et al., 2020 [8]. According to Leonti, 2022 [1], we used an approach mainly based on the analysis and discussion of primary data (how many times the species was cited for a given use sector and/or use category, including the used parts and the form of preparation/administration). The Mann–Whitney U test was performed to compare ethnobotanical knowledge in male and female informants, while the Kruskal–Wallis test was used to investigate possible differences among the studied communities. Spearman correlation analysis was used to test the relationship between the number of mentioned plants and informant age groups. Plant identification was performed with the support of botanists and naturalists from the research group and local mid-mountain guides. The botanical name was also reported according to both https://www.worldfloraonline.org (last accessed 6 February 2024) and Flora d’Italia [12]. Plant species were sometimes mentioned by the informants according to their own folk system. In this case, for *A. erba-rotta* subsp. *moschata*, the phenomenon of over-differentiation was documented, both during the fieldwork and in building the database, and every different local name given by the *malenca* people to identify this plant species was accurately recorded [50,51].

### 3.2. Plant Material

For the pharmacological investigations, the flower heads of *A. erba-rotta* subsp. *moschata* were gathered in July 2021 in Alpe Mastabbia, at 2200 m a.s.l. (Chiesa in Valmalenco, Sondrio, Lombardy, Italy). The species grows wild on siliceous substrates and niches with accumulations of raw humus, at windy and exposed ridges. Voucher specimens were labeled with the codes GBG123/GBG124 and deposited in the Herbarium of the Ghirardi Botanical Garden of the University of Milan (DISFARM, Toscolano Maderno, Brescia, Italy). Prof. G. Fico and Prof. C. Giuliani identified the species according to Pignatti et al. [12].

### 3.3. Molecular Docking Analysis

Molecular docking analyses were performed using the isoform TAS2R46, belonging to the TAS2R Bitter Taste Receptors family (experimentally resolved by cryo-EM in 2022, PDB ID 7XP6) [41], since it is the only resolved structure belonging to this family. The structure was employed to perform molecular docking analyses, with the aim to predict the preferred conformation of the 31 phytochemicals identified for *A. erba-rotta* subsp. *moschata* in Bottoni et al. [13] within the binding site of the bitter taste receptor to obtain the most stable complex. The predicted binding affinity was given by a scoring function and each used software program had its own search algorithm and specific scoring functions.

The experiment started with the protein structure preparation. The force field CHARMM22 and Gasteiger’s charges were assigned, and the H++ server was chosen to predict the protonation state of ionizable protein groups. The protein was then refined by 2 minimization procedures using NAMD. The first procedure was carried out with all the protein atoms fixed except for hydrogens; the second one was performed with the backbone atoms under constraints to preserve the resolved folding. The thus prepared protein structure underwent the following docking simulations.

Firstly, a redocking study of the co-crystallized strychnine within the TAS2R46 pocket was performed to evaluate the accuracy of the two molecular docking algorithms in terms of the capacity to rebuild the experimental pose. A binding pocket of 9Å around the co-crystallized strychnine was considered. The protein was simulated rigid, and the ligand was considered flexible. The software programs used were v. 1.2 PLANTS (algorithm: ACO—Ant Colony Optimization) [52] and v. 5.8.1 GOLD (algorithm: GA—Genetic Algorithm) [53]. In PLANTS docking, the ChemPLP scoring function and speed1 were set, while, in GOLD docking, the ChemPLP scoring function and very flexible option for the search efficiency were set. Ten poses for each docking study were generated.

Finally, molecular docking studies were conducted for all the identified compounds for *A. erba-rotta* subsp. *moschata* in Bottoni et al. [13], considering the same settings of the redocking procedure, and a consensus analysis between the two sets of results from the two docking algorithms was performed. Molecules were ranked from the best one to the worst one and the consensus score was calculated as the sum of the two ranking positions. In the event of a lack of specific positional or conformational isomers in Bottoni et al. [13], those tested and detailed in the table refer to previous compound identification studies performed on *A. erba-rotta* subsp. *moschata* derivatives.

### 3.4. Pharmacological Investigation

#### 3.4.1. Preparation of the Extracts

For the pharmacological investigation, inflorescences of *A. erba-rotta* subsp. *moschata* were harvested in Valmalenco (Alpe Mastabbia, 2200 m a.s.l.). The sample was air-dried, and three types of extraction were performed: infusion, decoction, and extraction with a mixture of ethanol and water 50:50 (hydroethanolic extraction). Infusion and decoction were chosen according to the primary data collected during the ethnobotanical investigations. During the interviews, the respondents reported that they primarily used hot aqueous extracts as the main preparation form. However, differences in the extraction times were observed; for this reason, we prepared both an infusion and decoction. For infusion preparation, 150 mL of deionized water was heated. Once boiling, it was poured over 3 g of plant material; the infusion was performed for 10 min and then the sample was filtered using Whatman filter paper. Decoction was carried out by placing 150 mL of deionized water onto 3 g of dried plant material. Once boiling, the heating was stopped, and the drug was left in the decoction for 20 min and then filtered. To compare the most traditionally used extracts with a hydroethanolic extract, 300 mL of ETOH:H_2_O (50:50) was poured over 3 g of the dried drug at room temperature. After 4 h incubation on a shaker at room temperature, the first filtration of the extract was performed. A second overnight extraction was conducted on the previously filtered drug using a shaker at room temperature with an additional 300 mL of the ETOH:H_2_O mixture (50:50). Then, the extract was filtered again and mixed with the previous one. The aqueous extracts were freeze-dried, while the hydroethanolic extract was firstly subjected to an alcohol evaporation phase through a rotary evaporator under reduced pressure at 37 °C (RE121, Buchi, Switzerland), and then freeze-dried. An aliquot of each extract was solubilized in H_2_O:DMSO (50:50), obtaining a concentration of 25 mg/mL; all aliquots were stored at −20 °C until treatment.

#### 3.4.2. Cell Culture

Gastric epithelial cells (GES-1) are human non-tumoral epithelial cells; they were provided by Dr. Dawit Kidane-Mulat (Howard University, College of Medicine, Washington, DC, USA). GES-1 cells were cultivated in RPMI 1640 medium (Gibco, Thermo Fisher Scientific, Waltham, MA, USA), supplemented with penicillin 100 units/mL, streptomycin 100 mg/mL, 1% L-glutamine 2 mM (Gibco, Thermo Fisher Scientific, Waltham, MA, USA), and 10% heat-inactivated fetal bovine serum (Euroclone S.p.A, Pero, Italy). Cells were incubated at 37 °C, 5% CO_2_, in a humidified atmosphere. Cells were detached by trypsin ethylenediamine-tetra-acetic acid (EDTA) 0.25% solution (Gibco, Thermo Fisher Scientific, Waltham, MA, USA) from a flask (Primo^®^, Euroclone S.p.A., Pero, Italy) every 72 h upon reaching confluency, and they were seeded in a new flask with fresh medium (1 × 10^6^ cells) for the following sub-culture.

Bacterial culture—*H. pylori* CagA-positive strain 26695 (ATCC 700392TM, VA, USA) was cultured in Petri dishes (Primo^®^, Euroclone S.p.A., Pero, Italy) containing Mueller–Hinton broth (Difco™, BD, Franklin Lakes, NJ, USA), 5% agar (Merck Life Science, Milan, Italy), and 25% defibrinated sheep blood (Thermo Fisher Scientific, Oxoid^TM^, Hampshire, UK), for 72 h, under a microaerophilic atmosphere (5% O_2_, 10% CO_2_ and 85% N2) at 37 °C and 100% humidity. Then, the bacteria were recovered from the Petri dishes and counted by optical density (OD) at 600 nm (OD value = 5 corresponds to about 2 × 10^8^ bacteria).

#### 3.4.3. Cell Treatments

GES-1 cells were seeded in a 24-well plate (Falcon^®^, Corning Life Science, Amsterdam, The Netherlands) at a density of 3 × 10^4^ cells/well or in a 12-well plate at a density of 6 × 10^4^ cells/well, according to the specific investigation. After 72 h, GES-1 cells were treated with the pro-inflammatory stimulus TNFα (10 ng/mL) or *H. pylori* (bacterium-to-cell ratio of 50:1) for 1 or 6 h, along with the extracts at different concentrations. In the co-culture, serum starvation was performed the day before the treatment to allow the synchronization of cells, using 0.5% serum medium, supplemented with 1% L-glutamine and 1% penicillin/streptomycin. The treatments in the co-culture approach were conducted with serum- and antibiotic-free medium, whereas TNFα treatments were carried out in serum-free medium. During the treatment, cells were maintained in an incubator at 37 °C and 5% CO_2_.

#### 3.4.4. Measurement of Anti-Inflammatory Activity

Measurement of IL-8 and IL-6 release—The pro-inflammatory mediators IL-8 and IL-6 were quantified in cell media after 6 h treatment with TNFα or *H. pylori* as stimuli and the extracts in 24-well plates, using a two-sandwich enzyme-linked immunosorbent assay (Human Interleukin-8 ABTS ELISA Development Kit and Human Interleukin-6 ABTS ELISA Development Kit, Peprotech, London, UK). The assay was conducted according to the manufacturer’s instructions, as previously reported [54]. The absorbance of the sample at 405 nm, 0.1 s (Victor^TM^ X3, PerkinElmer, Waltham, MA, USA) was compared to the absorbance of human recombinant standard IL-8 (0–1000 pg/mL). EGCG (20–50 μM) was used as a reference compound. Data (mean ± SEM of at least three experiments) were expressed as a percentage relative to stimulated controls, which were arbitrarily assigned the value of 100%.

NF-κB activation—GES-1 cells were transiently transfected with 50 ng/well of a reporter plasmid, in which the luciferase gene was under the control of the E-selectin promoter containing three κB elements responsive to NF-κB, in 24-well plates. Lipofectamine^®^ 3000 reagent (Invitrogen, Thermo Fisher Scientific, Waltham, MA, USA) was used for the transfection, according to the manufacturer’s instructions. The plasmid was a gift from Dr. Marx (Department of Internal Medicine—Cardiology, University of Ulm; Ulm, Germany). The day after transfection, cells were treated with TNFα or deactivated *H. pylori* (5 min, 95 °C) and the extracts for 6 h. At the end of the treatment, the luciferase activity was measured using Britelite^TM^ Plus reagent (Perkin Elmer, Milan, Italy), according to the manufacturer’s instructions. Apigenin (20–50 μM) was used as a reference compound. Data (mean ± SEM of at least three experiments) were expressed as a percentage relative to stimulated controls, which were arbitrarily assigned the value of 100%.

#### 3.4.5. Measurement of Antibacterial Activity

Measurement of *H. pylori* growth—The microbroth dilution method was performed as per the recommendations of the Clinical and Laboratory Standards Institute (CLSI) [55] and was used to determine the minimum inhibitory concentration (MIC). Extracts at different concentrations were prepared in 5% FBS Brucella broth (BBL™, BD, Franklin Lakes, NJ, USA), and 100 μL of each sample was placed in a 96-well U-bottom plate (Greiner Bio- One™, Rome, Italy). Then, 100 μL of *H. pylori* suspension (OD = 0.1) prepared in the same medium was added to each well. After mixing, the 96-well plate was incubated at 37 °C in a 5% CO_2_ incubator under microaerophilic conditions. After 72 h, the assay plate was read visually for growth inhibition at 600 nm, 0.1 s, using a multi-detection microplate reader (Victor™ X3, PerkinElmer, Waltham, MA, USA). Tetracycline (0.125 μg/mL) was used as a reference compound. Data (mean ± SEM of at least three experiments) were expressed as a percentage relative to *H. pylori* growth alone, which was arbitrarily assigned the value of 100%.

Measurement of *H. pylori* adhesion to cells—The bacterial adhesion was measured using a cytofluorimetric method adapted from Messing et al. [56] and reported in Piazza et al. [24]. GES-1 cells, in 12-well plates, were infected with CFSE-labeled *H. pylori*. CFSE solution (carboxyfluorescein succinimidyl ester (CFSE) 5 mM, CellTrace^TM^, Cell Proliferation Kits, Invitrogen, Thermo Fisher Scientific, Waltham, MA, USA) was added to suspended bacteria (2 μL of CFSE each 108 bacteria) and incubated for 45 min at 37 °C; then, the bacterial suspension was centrifuged (3150× *g*, 5 min) and washed two times with PBS 1× to remove the excess probe. After 1 h treatment, cells were washed two times with PBS 1×, collected through a scraper in PBS/EDTA 2 mM, and centrifuged (300× *g*, 5 min); then, they were fixed with formaldehyde (4% in PBS 1×) and incubated in an ice bath for 10 min. Finally, cells were centrifuged, washed, and resuspended in 0.5% BSA (PBS/EDTA 2 mM) for analysis, using the NovoCyte flow cytometer (ACEA Biosceinces, San Diego, CA, USA) and NovoExpress 1.3.0 software (ACEA Biosciences). Procyanidin A2 (500 μM) was used as a reference compound. Data (mean ± SEM of at least three experiments) were expressed as a percentage relative to *H. pylori*’s maximum adhesion to cells, which was arbitrarily assigned the value of 100%.

#### 3.4.6. Cell Viability

The integrity of the cell morphology was verified by light microscope inspection before and after treatment. Cell viability was assessed after 6 h treatment, using the 3-(4,5-dimethylthiazol-2-yl)-2-5-diphenyltetrazolium bromide (MTT, Merck Life Science, Milan, Italy) method [57]. This method evaluates the activity of a mitochondrial enzyme, the succinate dehydrogenase, as an index of viability. Briefly, the medium was discarded; then, 200 μL of MTT solution (0.1 mg/mL in PBS 1×) was added to each well and they were kept in darkness (45 min, 37 °C). Then, the MTT solution was discarded and the purple salt included in the cells was dissolved by isopropanol:DMSO (90:10 *v*/*v*), and the absorbance was read at 595 nm (Victor^TM^ X3, Perkin Elmer, Walthman, MA, USA).

#### 3.4.7. Statistical Analysis

All biological results were expressed as the mean ± SEM of at least three independent experiments. Data were elaborated through an unpaired ANOVA test and Bonferroni post-hoc analysis. Statistical assessment and IC_50_ calculation were conducted using the GraphPad Prism 9.0 software (GraphPad Software Inc., San Diego, CA, USA). Values of *p* < 0.05 were considered statistically significant.

## 4. Conclusions

This work represented a further effort to deepen the overall quali-quantitative primary data documented in Valmalenco specifically for *A. erba-rotta* subsp. *moschata*, to obtain even more absolute information on the species’ potential for the treatment of dyspepsia disorders. In the study, men and women shared the same cultural heritage regarding the health properties of the species, and its inflorescences’ collection, passed down for generations, expressed a sense of territorial belonging to the *malenca* community. The local folk medicine described the decoction of the plant’s inflorescences as a strong stomachic and eupeptic, and for stomach pain, sometimes associated with gastrointestinal ailments. According to popular belief, the health benefits of the plants are reflected in its strong bitter taste. Coherently with this, a molecular docking analysis of the plant’s polyphenolic profile highlighted the phytocomplex’s potential to accommodate the TAS2R46 bitter taste receptors. This process provided an element of novelty among the other studies on this plant species. Along with the pharmacological investigations of three different extracts from *A. erba-rotta* subsp. *moschata*, our findings confirmed the traditional use of the plant. In particular, the aqueous extracts (infusion and decoction) showed anti-inflammatory effects, both inhibiting TNFα-induced IL-8 and IL-6 release and the NF-κB pathway. Their efficacy was even better when the stimulus was *H. pylori* and involved the inhibition of the pathogen’s growth and adhesion to the gastric epithelium. This evidence could contribute to the treatment of gastric ailments and the discomfort typical of *H. pylori*-induced dyspepsia. The ethnobotanical indications regarding the moderate consumption of *A. erba-rotta* subsp. *moschata* products may be associated with the low active concentrations of the extracts (i.e., μg/mL range). However, we could not rule out that the aqueous preparations may act as anti-inflammatory and anti-*H. pylori* agents at low concentrations and increase gastric juice production at higher concentrations, due to their bitterness. This may explain the traditional recommendations raised during the interviews. Future studies will be conducted to identify which components most retain the biological effects herein observed and investigate whether they are the same as those that exhibit a bitter taste and an interaction with the TAS2R receptors.

## Figures and Tables

**Table 1:** Partitioning by gender and age of the pool of informants. Results of the Mann–Whitney U and Spearman tests are shown considering the overall data of the ethnobotanical survey.

Gender	Number of Informants	All Uses Mean ± sd.	Medicinal Uses Mean ± sd.
Female	224	1.81 ± 0.73	1.07 ± 0.50
Male	107	1.77 ± 0.75	0.98± 0.41
U Mann–Whitney Test:		U = 11683.6; Z = 0.337; *p* = 0.74	U=11172.0; Z= 1.475; *p* = 0.14
Age Groups	Number of Informants	All Uses Mean ± sd.	Medicinal Uses Mean ± sd.
FROM 20 TO 30	19	1.74 ± 0.56	0.89 ± 0.32
FROM 31 TO 40	38	1.66 ± 0.74	0.89 ± 0.31
FROM 41 TO 60	93	1.70 ± 0.70	0.99 ± 0.40
FROM 61 TO 80	141	1.82 ± 0.64	1.05 ± 0.47
OVER 80	40	2.07 ± 0.85	1.30 ± 0.72
Spearman Test:		R = 0.131; *p* < 0.05	R = 0.135; *p* < 0.05
Communities	Number of Informants	All Uses Mean ± sd.	Medicinal Uses Mean ± sd.
CAS	111	1.74 ± 0.56	1.08 ± 0.50
CHI	116	1.78 ± 0.81	1.12 ± 0.50
LAN	69	1.76 ± 0.55	0.85 ± 0.46
SPR	10	2.20 ± 1.03	1.00 ± 0.00
TOR	25	1.80 ± 0.64	0.96 ± 0.20
Kruskal–Wallis Test:		H = 2.901; *p* = 0.574	H = 13.134; *p* < 0.001

**Table 3:** IC_50_ values (μg/mL) of the *A. erba-rotta* subsp. *moschata* extracts on IL-8 and IL-6 release and NF-κB activation.

*A. erba-rotta* subsp. *moschata* W. extracts vs. TNFα	IC_50_ (μg/mL)		
	IL-8 Inhibition	IL-6 Inhibition	NF-κB Inhibition
Infusion	178.1	34.8	81.29
Decoction	>200	85.77	89.51
Hydroethanolic extract	123.7	33.31	52.09
***A. erba-rotta*** ** subsp. *moschata* W. extracts vs. *H. pylori***	**IC_50_ (μg/mL)**		
	**IL-8 Inhibition**	**IL-6 Inhibition**	**NF-** **κB Inhibition**
Infusion	60.71	45.04	57.2
Decoction	60.05	43.8	57.17
Hydroethanolic extract	38.03	28.94	59.65

## Data Availability

This paper contains all data collected by the authors on *A. erba-rotta* subsp. *moschata*. Complete data from the interviews are unavailable due to privacy restrictions.

## Supplementary Materials

The following supporting information can be downloaded at: https://www.mdpi.com/article/10.3390/plants13040539/s1, Figure S1. Cell viability (MTT test). Table S1. Raw primary data recorded during the interviews: translation in English and original Italian sentence.
