# Supplementary material for: From Primary Data to Ethnopharmacological Investigations on Achillea erba-rotta subsp. moschata (Wulfen) I.Richardson as a Remedy against Gastric Ailments in Valmalenco (Italy)
Source: Plants (Basel). 2024 Feb 16;13(4):539. doi: 10.3390/plants13040539 (PMC10891827; doi:10.3390/plants13040539)
Supplement: Supplementary file 1 [file plants-13-00539-s001.zip › plants-2813559-supplementary.pdf]

SUPPLEMENTARY MATERIAL

From Primary Data to Ethnopharmacological Investigations on *Achillea erba-rotta* subsp. *moschata* (Wulfen) I.Richardson as a Remedy against Gastric Ailments in Valmalenco (Italy)

Martina Bottoni [1,2], Giulia Martinelli [3], Nicole Maranta [3], Emanuela Sabato [1], Fabrizia Milani [1,2,*], Lorenzo Colombo [1,2], Paola Sira Colombo [1,2], Stefano Piazza [3], Enrico Sangiovanni [3], Claudia Giuliani [1,2], Piero Bruschi [4], Giulio Vistoli [1], Mario Dell'Agli [3] and Gelsomina Fico [1,2]

1 Department of Pharmaceutical Sciences, University of Milan, Via Mangiagalli 25, 20133 Milan, Italy; martina.bottoni@unimi.it (M.B.); emanuela.sabato@unimi.it (E.S.); lorecolo.93@gmail.com (L.C.); pasico19@virgilio.it (P.S.C.); claudia.giuliani@unimi.it (C.G.); giulio.vistoli@unimi.it (G.V.); gelsomina.fico@unimi.it (G.F.)

2 Botanical Garden G.E. Ghirardi, Department of Pharmaceutical Sciences, University of Milan, Via Religione 25, 25088 Toscolano Maderno (BS) Italy

3 Department of Pharmacological and Biomolecular Sciences "Rodolfo Paoletti", University of Milan, Via Balzaretti 9, 20133 Milan, Italy; giulia.martinelli@unimi.it (G.M.); nicole.maranta@unimi.it (N.M.); stefano.piazza@unimi.it (S.P.); enrico.sangiovanni@unimi.it (E.S.); mario.dellagli@unimi.it (M.D.)

4 Department of Agricultural, Environmental, Food and Forestry Science and Technology, University of Florence, Piazzale delle Cascine 18, 50144 Florence, Italy; piero.bruschi@unifi.it

* Correspondence: fabrizia.milani@unimi.it

Figure S1. *Cell viability (MTT test).* GES-1 cells were treated for 6 h with TNFα (10 ng/mL) (panel **A**) or *H. pylori* (ratio 50:1, bacteria:cell) (panel **B**). Results were expressed as mean ± SEM (at least three experiments) of the relative % in comparison to stimulus (black bar), to which the value of 100% was arbitrarily assigned. I, *A. erba-rotta* subsp. *moschata* infuse; D, *A. erba-rotta* subsp. *moschata* decoction; HE, *A. erba-rotta* subsp. *moschata* hydroethanolic extract.

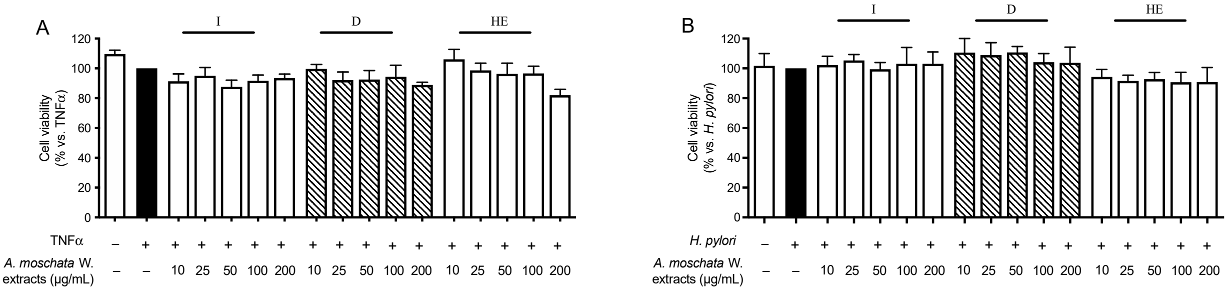

Table S1: Raw primary data recorded during the interviews: translation in English and original Italian sentence.

English	Italian
With that, it either goes up or down	*Con quella, o va su o va giù*
It is a lifesaver	*È un salvavita*
It is extraordinary	*È portentosa*
It is very bitter and powerful	*È amarissima e molto potente*
The bitter taste is the good effect	*L'amaro è l'effetto buono*
It is a medicinal herb, 3-4 flowers and it helps me digest	*È un'erba medicinale, 3-4 fiorellini e mi fa digerire*
It never hurts to drink it	*Non fa mai male berla*
However, you must not overdo it, too many flowers cause stomach-ache	*Non bisogna esagerare, troppi fiori provocano mal di pancia*
Very few flowers are enough to have an effect	*Bastano davvero pochi fiori per fare effetto*
It causes me tachycardia	*A me provoca tachicardia*
I don't know the *erba iva*, I know the *daneda*	*Non conosco l'*erba iva*, io conosco la* daneda
Everyone uses it in Valmalenco	*Tutti la usano qui*
Erba iva has never been lacking in our house	*L'*erba iva *non è mai mancata in casa nostra*
This is the manner here, you have to bring them together and let them dry	*Qui si fa così, devi riunirli e farli essiccare*
The plant should never be torn off	*La pianta non va mai strappata*
You have to cut it more or less at this height, moving the flowers	*Devi tagliarla più o meno a questa altezza, spostando i fiori*
You must always leave the roots, if you want to find it again next year	*Lascia sempre le radici, se vuoi ritrovarla l'anno prossimo*
Don't pick it up here, my neighbor always comes here	*Qui non raccoglierla, ci viene sempre il mio vicino*
It was the treasure that made survival possible	*Era il tesoro che permetteva di vivere*
